# USP50 regulates NLRP3 inflammasome activation in duodenogastric reflux-induced gastric tumorigenesis

**DOI:** 10.3389/fimmu.2024.1326137

**Published:** 2024-02-26

**Authors:** Chenye Zhao, Mingchao Mu, Xiaopeng Li, Zepeng Dong, Jiahao Wang, Chengzhi Yao, Jianbao Zheng, Xuejun Sun, Junhui Yu

**Affiliations:** Department of General Surgery, The First Affiliated Hospital of Xi’an Jiaotong University, Xi’an, Shaanxi, China

**Keywords:** USP50, gastric tumorigenesis, NLRP3 inflammasome, pyroptosis, HMGB1

## Abstract

Duodenogastric reflux (DGR) has been linked to the onset of gastric cancer (GC), although the precise mechanism is yet obscure. Herein, we aimed to investigate how refluxed bile acids (BAs) and macrophages are involved in gastric carcinogenesis. In both active human bile reflux gastritis and the murine DGR model, ubiquitin specific protease 50 (USP50) was dramatically raised, and macrophages were the principal leukocyte subset that upregulated USP50 expression. Enhancing USP50 expression amplified bile acid-induced NLR family pyrin domain containing 3 (NLRP3) inflammasome activation and subsequent high-mobility group box protein 1 (HMGB1) release, while USP50 deficiency resulted in the reversed alteration. Mechanistically, USP50 interacted with and deubiquitinated apoptosis-associated speck-like protein containing CARD (ASC) to activate NLRP3 inflammasome. The release of HMGB1 contributes to gastric tumorigenesis by PI3K/AKT and MAPK/ERK pathways. These results may provide new insights into bile reflux-related gastric carcinogenesis and options for the prevention of DGR-associated GC.

## 1 Introduction

Gastric carcinoma (GC) is a widespread and life-threatening type of gastrointestinal malignancy on a global scale (1). The initiation of gastric carcinoma (GC), particularly the intestinal subtype, typically arises from persistent gastric inflammation, atrophic gastritis, and intestinal metaplasia (IM). Gastric stump carcinoma (GSC), which emerges following gastric surgery of either benign or malignant conditions, is now extensively acknowledged as a distinctive subtype of GC. The stump carcinoma frequently occurs at the anastomosis, a region susceptible to severe duodenogastric reflux (DGR). As the major component of duodenal juice, bile acids have been linked to gastric carcinogenesis following gastrectomy in clinical data and animal studies (2). Our previous study revealed that upon deoxycholic acid (DCA) exposure, normal gastric mucosa transformed into IM, which is considered an “irreversible point” in gastric carcinogenesis (3). Nevertheless, there has been contention regarding the contribution of DGR as a significant causative factor in gastric carcinogenesis for numerous decades, as the precise mechanism linking DGR to the onset of gastric cancer still eludes our understanding.

The innate immune system possesses the ability to discern microbes or endogenous molecules referred to as damage-associated molecular patterns (DAMPs) or pathogen-associated molecular patterns (PAMPs) through host pattern recognition receptors (PRRs) (4). Activation of PRRs can initiate inflammatory signaling transduction cascades, triggering inflammation. NLR family pyrin domain containing 3 (NLRP3) inflammasome, the most fully comprehensively characterized inflammasome, is triggered by multiple PAMPs or DAMPs in the inflammatory response (5). Upon ligand sensing, NLRP3 and apoptosis-associated speck-like protein containing CARD (ASC) collaborate to create a platform that facilitates the recruitment and activation of caspase-1 (6). Active caspase-1 catalyzes the maturation and release of IL-1β/18 and drives pyroptosis by cleaving gasdermin D (GSDMD) (7). Emerging evidence indicated that NLRP3 inflammasome is implicated in the development of various inflammatory disorders (8, 9). Moreover, in tumorigenesis, NLRP3 inflammasomes have been demonstrated to exert important, albeit contrasting, roles (10). Pyroptosis derived by NLRP3 inflammasome acts as a proinflammatory role in multiple human inflammatory diseases (11, 12). However, NLRP3 inflammasome-mediated pyroptosis in human bile reflux gastritis has not been comprehensively elucidated.

Bile acids (BAs) comprise a variety of amphipathic compounds formed within hepatocytes through cholesterol metabolism, subsequently discharged into the intestinal tract, and subjected to additional metabolism and alterations facilitated by the gut microbiota. Bile acids play pivotal roles not only in lipid digestion, absorption of fat-soluble vitamins, cholesterol metabolism, and bile excretion, but also in modulation of gut microbiota and regulation of glucose metabolism (13). Intriguingly, emerging research has illuminated that bile acid signaling contributes to immunological homeostasis via both innate and adaptive immune responses, functioning as the pro-inflammatory or anti-inflammatory role (14). Che et al. (15) found that bile acids acted as DAMPs to excite the activation of NLRP3 inflammasome and led to pyroptosis in macrophages under cholestatic conditions. Another study revealed DCA could activate the NLRP3 inflammasome, promote the polarization of M2 macrophages, and aggravate intestinal tumorigenesis in APC^min^/^+^ mice (16). It remains enigmatic whether bile acids, under DGR conditions, promote gastric tumorigenesis via NLRP3 inflammasome activation.

Ubiquitination serves as a crucial post-translational modification governing vital cellular processes, encompassing transcription, signal transduction, cell cycle, DNA-damage repair, and development (17). Ubiquitination is achieved by an E1, E2, and E3 enzymatic cascade that covalently attaches ubiquitin to the substrate proteins. Deubiquitinating enzymes (DUBs) can antagonize the process of ubiquitination by interacting directly or indirectly with substrates to remove ubiquitin (18). Ubiquitin specific protease 50 (USP50), a member of the USP family, has been identified as a novel human DUB induced in response to DNA damage. USP50 engages with HSP90 and modulates the protein abundance of Wee1, a pivotal element in G2/M cell cycle arrest (19). However, few studies focus on the role of USP50 on bile reflux gastritis and GC (20).

Herein, our results uncover a previously unappreciated phenomenon coupling USP50 to DGR-associated gastric tumorigenesis. Mechanistically, USP50 accelerates bile acid-induced NLRP3 inflammasome activation and pyroptosis via interacting with and deubiquitinating ASC in macrophages. Subsequent high-mobility group box protein 1 (HMGB1) release from pyroptotic macrophages contributes to gastric tumorigenesis by PI3K/AKT and MAPK/ERK pathways.

## 2 Materials and methods

### 2.1 Clinical specimen

Patients diagnosed with bile reflux gastritis (BRG) or gastric cancer (GC) by gastric endoscopy, biopsy, or surgery from 2018 to 2021 in the First Affiliated Hospital of Xi’an Jiaotong University were screened and qualified specimens were collected. Informed consents were signed by all participants.

### 2.2 Mice and animal surgery

6-8 weeks old C57BL/6 male mice were purchased from the Animal Experimental Center of Xi’an Jiaotong University and maintained in an SPF environment with 12 h light/dark cycle at 20–22°C. Twenty mice were distributed to the Sham group or gastrojejunostomy group (GJ) randomly (10 mice in each group). Before the surgery, mice were anesthetized via isoflurane inhalation. For sham surgery, an incision was performed on the greater curvature of the stomach and sutured soon. For GJ surgery, the procedure was performed as described previously (21). Briefly, an approximate 0.3 cm incision was performed on the greater curvature and proximal jejunum respectively, and side-to-side anastomosis was constructed to establish the DGR model. Postoperatively, animals were provided water and moist chow 24 hours following surgery. After 6 months, mice fasted for 12 hours were euthanized by cervical dislocation. The specific tissues were collected. All animal experiments were approved by the Animal Care and Use Committee of the Xi’an Jiao Tong University.

### 2.3 Cell culture

HGC27, SNU216, RAW264.7, U937, and 293T were purchased from the Chinese Academy of Sciences Cell Bank (Shanghai, China). Cells were cultured with RPMI 1640 with 10% FBS and 1% penicillin and streptomycin in a humidified environment containing 5% CO2 at 37°C. PMA (P1585, Sigma-Aldrich) was added to the culture medium with a final concentration of 10 ng/ml for 48 hours to induce the differentiation of U937 towards macrophage. LPS (Sigma-Aldrich) at 1 µg/mL was used to stimulate differentiated U937 or RAW264.7 cells. Subsequently, TDCA was added to the medium with a specific concentration and duration. Conditioned media (CM) was acquired by the collection of supernatants of macrophages with specific treatment after centrifugation.

### 2.4 Drugs and reagents

Taurodeoxycholic Acid (TDCA) (GC44995, GLPBIO, USA) was dissolved in DMSO and then added to the medium. Recombinant human HMGB1 protein (rhHMGB1) (ab285780, Abcam) at 100 ng/mL for 24 h, and anti-HMGB1 neutralizing antibodies (HMGB1 NAs) (ab79823, Abcam) were used to mimic or to counter the secreted HMGB1. Lipopolysaccharide (LPS) and MG132 were purchased from Sigma-Aldrich (St. Louis, MO, USA). SCH772984 (S7101, Selleck) and MK-2206 (S1078, Selleck) were used as ERK1/2 and AKT inhibitors respectively.

### 2.5 Western blotting

Total protein was obtained with RIPA lysis buffer (P0013B, Beyotime, Shanghai, China). BCA Protein Assay Kit (P0012, Beyotime, Shanghai, China) was employed to measure protein concentration. Proteins were subjected to SDS-page for separation and then transferred to PVDF membranes (0.22 µm, Millipore). NcmBlot blocking buffer (P30500, NCM Biotech, Soochow, China) was used to block nonspecific binding sites. The membranes were incubated with specific primary antibodies overnight at 4°C. Subsequently, washed membranes were incubated with corresponding secondary antibodies for 1 h at room temperature. ECL Chemiluminescence kit was purchased from Mishu Biotechnology (MI00607B, Xi ‘an, China). The primary antibodies included USP50 (24817-1-AP, Proteintech, USA), NLRP3 (ab263899, Abcam, UK), Caspase-1 (22915-1-AP, Proteintech, USA), GSDMD (#97558, CST, USA), IL-1β (#12242, CST, USA), IL-18 (10663-1-AP, Proteintech, USA), ASC (ab309497, Abcam, UK), Ub (10201-2-AP, Proteintech, USA), p-AKT (66444-1-Ig, Proteintech, USA), AKT (10176-2-AP, Proteintech, USA), ERK1/2 (ab184699, Abcam, UK), p-ERK1/2 (# 4370S, CST, USA), HMGB1 (ab18256, Abcam, UK) and GAPDH (60004-1-Ig, Proteintech, USA). The second antibodies included goat anti-mouse IgG-HRP (abs20039, Absin, China) and goat anti-rabbit IgG-HRP (abs20040, Absin, China).

ASC oligomerization was performed as previously described (22). In short, cells differentiated by PMA were stimulated with LPS first. Next, cells received specific treatments. Cells were lysed with NP-40 lysis buffer and centrifugated. After removal of the supernatant, the insoluble cell fragments were resuspended in ice cold-PBS containing 2 mM DSS (Sigma−Aldrich, USA) and incubated at 37°C for 30 minutes to allow for crosslinking of ASC oligomers. Centrifuged and washed samples to remove the unbound DSS. The precipitate was resuspended in 1×loading buffer for western blotting detection.

### 2.6 Lentiviral transduction and plasmid construction

USP50 overexpression and knock-down were achieved by lentiviral vectors supplied by Gene Chem Co., Ltd. (Shanghai, China). Lentiviral infection was performed according to the manufacturer’s protocol.

HA-ubiquitin plasmid and its mutant (K48R, K63R) plasmid constructed by cloning the corresponding DNA sequence into the pCMV-HA vector were purchased from Gene Chem Co., Ltd. (Shanghai, China). Lipofectamine 3000 reagents (L3000008, Invitrogen, USA) were applied to transfected plasmids into objective cells according to the manufacturer’s protocols.

### 2.7 Measurement of bile acids

The stomach contents were collected at once after the mice were sacrificed and shipped to Majorbio company (Shanghai, China) at -80°C for analysis of bile acids. The instrument platform for LC-MS (Liquid Chromatography-Mass Spectrometry) analysis is UHPLC-Q Exactive system of Thermo Fisher Scientific.

### 2.8 Measurement of ELISA and ATP

Human IL-18 ELISA Kit (ab215539), Mouse IL-18 ELISA Kit (ab216165), Human IL-1β ELISA Kit (ab214025), Mouse IL-1β ELISA Kit (ab197742), Mouse S100A8 ELISA Kit (ab263886), Mouse S100A9 ELISA Kit (ab213887) and HSP70 ELISA Kit (ab133060) were purchased from Abcam, UK. Human S100A8 (E-EL-H1289c), Human S100A9 (E-EL-H1290c), HSP90 ELISA Kit (E-EL-H1864c), Human HMGB-1 ELISA Kit (E-EL-H1554c), Mouse HMGB-1 ELISA Kit (E-EL-M0676c) were obtained from Elabscience Biotechnology Co., Ltd, China. ATP Assay Kit (S0027) was purchased from Beyotime, China.

Briefly, differentiated U937 or RAW264.7 was stimulated with LPS for 6 h. Next, TDCA was added to the medium at the final concentration of 200 μM for 24 h. Subsequently, the supernatant was collected to measure the level of DAMPs including HSP70, HSP90, S100A8, S100A9, and ATP. All assays were performed following the instructions of the producer.

### 2.9 co-IP

For co-immunoprecipitation (co-IP), 1x10^7^ cells were lysed by Cell lysis buffer for Western and IP (P0013, Beyotime, Shanghai, China) pre-mixed with PMSF and phosphatase inhibitors. After centrifugation, the supernatant was collected and mixed with IP grade ANTI-FLAG antibody (F1804, Sigma-Aldrich) or His antibody (ab18184, Abcam). Subsequently, the mixture was incubated overnight on a shaker buffer at 4°C. Agarose was added to the mixture and incubated for 4 h the following day. The protein-protein-agarose complexes precipitates were collected by centrifugation and washed three times. Finally, the co-IP products boiled with loading buffer were subjected to western blotting.

### 2.10 ASC speck assay

U937 cells transferred with an empty vector (shNC) or shUSP50 vector were differentiated by PMA and stimulated with LPS. TDCA was added to the medium for 24 hours. Rinse cells twice with PBS to remove the cell culture medium and then fix cells with 4% polyformaldehyde. Permeabilize cells in PBS + 0.1% Triton^®^ X-100 and block non-specific sites with PBST including 1% BSA+22.52 mg/mL glycine. Next, cells were incubated with ASC anti-body overnight at 4°C. Fluorescence-labeled secondary antibody was used to visualize ASC speck, and DAPI (C0065, Solarbio, China) was used to mark the nuclei.

### 2.11 Real-time quantitative PCR

Total RNA was extracted by the Total RNA extraction kit (DP419, TIANGEN, China) referred to as the manufacturer’s manual. Reverse transcription was performed in 20 μl reaction reagents of the RT Mix Kit (AG11728, Accurate Biotechnology, China). RealStar Fast SYBR qPCR Mix (A301-5, GenStar, China) was utilized to perform amplification reaction on the CFX96 Real-Time PCR Detection System (Bio-Rad, USA). GAPDH was used as the loading control. Primers were listed in **Supplementary Table 1**.

### 2.12 CCK-8

Cell counting Kit-8 (CCK8, CAT#GK10001) was obtained from GLPBIO company, USA. 2000 cells/well were seeded in 96-well plates. After 24 h, the normal medium was substituted with conditioned media (CM). CCK-8 reagent was added to each well to estimate cell counts at 24, 48, 72, and 96 h after seeding. Incubated for 2 h at 37°C, 96-well plates were subjected to a microplate reader to determine OD450 values.

### 2.13 Transwell assay

Transwell plates (Corning, USA) with Matrigel (BD, USA) were utilized to evaluate cell invasion abilities. Briefly, 8 ×10^4^ cells were seeded at the upper chamber, the lower chamber was filled with corresponding conditioned media (containing 20% FBS). After incubation for 24 h, cells on the upper surface of the Transwell plates were removed while invading cells on the underside were fixed and stained with crystal violet. Three fields of each Transwell plate were captured with an inverted microscope randomly for calculating cell numbers.

### 2.14 Scratch assay

Gastric cancer cells were seeded in six-well plates. When the cells reached full confluence, wounds were scratched with 10 μl pipette tips. Then the wells were rinsed with PBS and added conditioned medium. Pictures of the wounds were taken at 0 h and 24 h. The scratch areas were measured by Image J software.

### 2.15 HE, immunohistochemical and immunofluorescence staining

Excised gastric tissues were fixed with paraformaldehyde, dehydrated with gradient alcohol, and embedded with paraffin. Sections at a thickness of 4 µm were used for hematoxylin-eosin staining (HE), Immunohistochemistry (IHC), and immunofluorescence (IF) staining analyses as previously described. Briefly, for IHC, sections were subjected to dewaxing, antigen retrieval, and endogenous catalase removal in sequence. Then rabbit serum was added to block non-specific sites. Primary antibodies including USP50 (24817-1-AP, Proteintech), NLRP3 (68102-1-Ig. Proteintech, China), Caspase-1 (22915-1-AP, Proteintech), IL-1β (#12242, CST, USA), HMGB1 (ab79823, Abcam, UK) were incubated with sections overnight at 4°C respectively. Corresponding second antibodies were incubated and then DAB chromogenic solution was used to visualize the specific protein. After labeling the nucleus with hematoxylin, sections were scanned with a Digital tissue section scanner (3DHISTECH). Similarly, for IF, sections that had been dewaxed, antigen repaired, and blocked in advance were incubated with two specific primary antibodies (USP50, CD68 (GB113150, Servicebio, China), caspase-1, NLRP3, IL-1β, IL-18). Subsequently, fluorescence-labeled secondary antibodies and DAPI (C0065, Solarbio, China) were incubated. Finally, the sections were subjected to a tissue section scanner.

Inflammation scores in the gastric mucosa were determined according to the criteria of Nolan et al. (23). Briefly, inflammatory cell infiltration of the gastric mucosa by mononuclear cells and neutrophils were scored as follows: 1, mild multifocal; 2, mild widespread or moderate multifocal; 3, mild widespread and moderate multifocal or severe multifocal; 4, moderate widespread; 5, moderate widespread and severe multifocal; and 6, severe widespread.

The immunoreactive score (IRS) of each field was calculated by multiplying the score for the extent of positive cells and the score for the staining intensity. The score of the extent of positively stained cells was gained based on the percentage of positively stained cells: 0–5% (0), 6–25% (1), 26–50% (2), 51–75% (3), and 76–100% (4). The staining intensity was scored as: negative (0), light brown (1), brown (2), and dark brown (3).

### 2.16 Statistical analysis

All data are displayed as mean ± standard error (SE). The statistical difference was evaluated by Student’s t-test or ANOVA with SPSS 22.0 software (IBM Inc). Unless particularly stated, the relative quantification in western blotting, immunofluorescence, immunohistochemistry, Transwell assay, and Scratch assay were analyzed by Image J software. *P < 0.05, **P < 0.01, and *** *P*<0.001 represent significant differences with incremental extent.

## 3 Results

### 3.1 Macrophage pyroptosis occurred in human bile reflux gastritis and in experimental models

**Figure 1:**
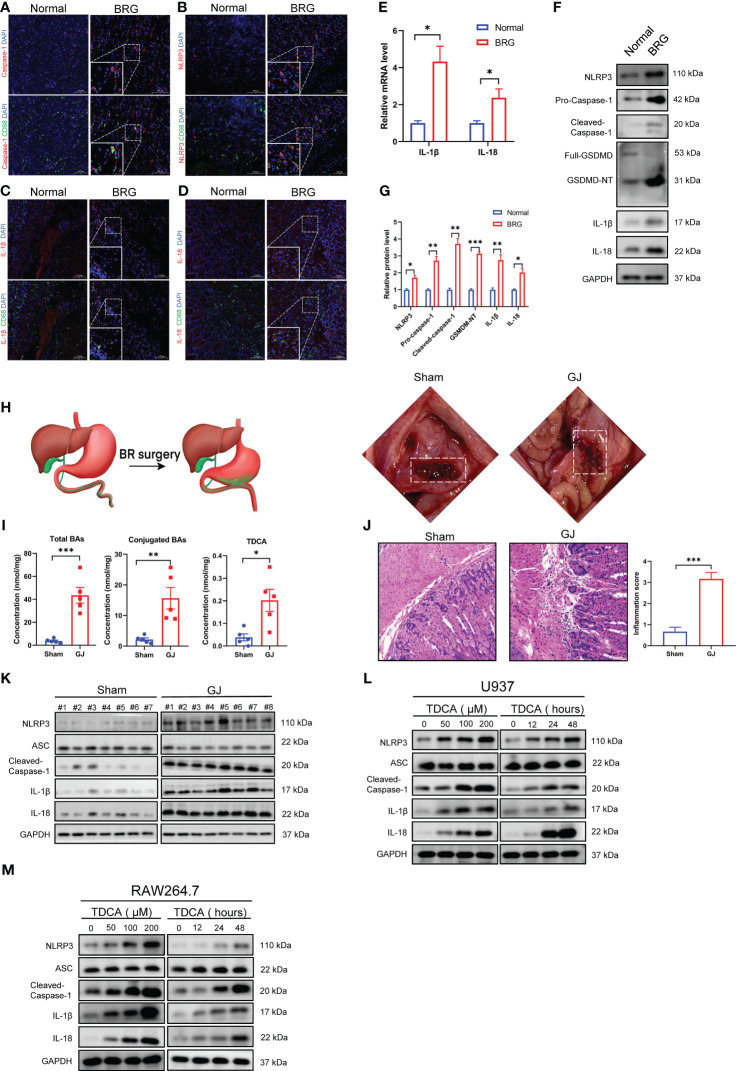
Macrophage pyroptosis occurred in human bile reflux gastritis and experimental models. **(A-D)** Caspase-1 **(A)**, NLRP3 **(B)**, IL-1β **(C)**, and IL-18 **(D)** were analyzed via Immunofluorescence staining (red) which was performed on paraffin sections of non-reflux normal tissues (Normal) and patients with bile reflux gastritis (BRG). CD68 was utilized to label macrophage (green) and nuclei were stained with DAPI (blue). **(E)** mRNA levels of IL-1β and IL-18 in normal tissues or tissues from BRG patients were evaluated by qRT-PCR. GAPDH is used as an internal reference. **(F, G)** Levels of pyroptosis-related proteins in Normal and BRG patient tissues were detected by western blotting and quantified by Image J software. GAPDH served as a loading control. **(H)** Schematic diagram of gastrojejunostomy (GJ) surgery in mice. **(I)** Total BAs, Conjugated BAs, and TDCA were measured by LC-MS. **(J)** Gastric mucosal inflammation triggered by DGR was evaluated by H&E staining in mice. **(K)** Pyroptosis-related proteins in murine gastric tissues were assayed with western blotting between the Sham and GJ groups. **(L, M)** Different concentrations (stimulated for 24 h) and durations (100μM TDCA) of TDCA were added to U937 **(L)** or RAW264.7 cells **(M)** as a DRG model *in vitro*, followed by the detection of pyroptosis-related markers through western blotting. *P < 0.05, **P < 0.01, and *** *P*<0.001.

First, this study investigated the expression of pyroptosis-related proteins in the stomachs of patients with bile reflux gastritis and the healthy population. In gastric mucosal biopsies from bile reflux gastritis patients, IF staining manifested that increased ‘scissors’ protein caspase-1 was coexpressed with CD68 in mucosa lamina propria (**Figure 1A**). NLRP3 and CD68 were also detected by IF (**Figure 1B**). As pyroptosis terminal cytokines which were cleaved from their precursor form by caspase-1, IL-1β and IL-18 levels were enhanced primarily in CD68+ macrophages in mucosal biopsies from bile reflux gastritis patients (**Figures 1C, D**). Additionally, compared with normal gastric tissues, the transcript levels of IL-1β and IL-18 were increased in bile reflux gastritis mucosa (**Figure 1E**). These results were further supported by western blotting analysis of pyroptosis-related proteins between gastric biopsies from bile reflux gastritis patients and normal tissues (**Figures 1F, G**). We further performed gastrojejunostomy (GJ) on C57BL/6 mice to establish the DGR model (**Figure 1H**). Consistent with the recent study (21), the GJ treatment significantly raised the levels of total BAs, conjugated BAs, and TDCA (tauroursodeoxycholic acid) in gastric contents (**Figure 1I**), which also demonstrated the bile reflux mouse model was well established. H&E analysis revealed that mice in the GJ surgery group experienced a startling chronic inflammation (**Figure 1J**). Consistent with our clinical study, the significantly elevated levels of pyroptosis-related proteins were delineated by IHC (**Supplementary Figure 1A**) and western blotting (**Figure 1K**; **Supplementary Figure 1B**). Additionally, the DGR model *in vitro* was conducted by treating of PMA-differentiated U937 and RAW264.7 macrophages with interval time or different dosages of TDCA exposure. Likewise, TDCA led to an increase in protein levels including NLRP3, active caspase-1, IL-1β, and IL-18 in a dose- and time-dependent manner (**Figures 1L, M**; **Supplementary Figures 1C-F**), while no alteration was observed in ASC level (**Figures 1L, M**). Collectively, these findings revealed a critical role for NLRP3 inflammasome-mediated macrophage pyroptosis in bile reflux gastritis.

### 3.2 The expression of USP50 is upregulated in bile reflux gastritis and GC

**Figure 2:**
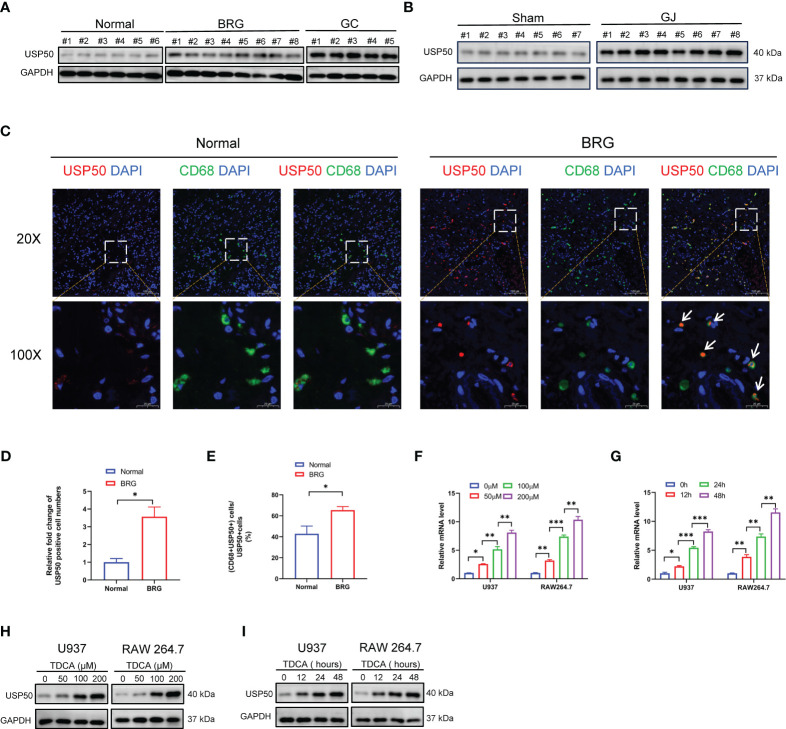
The expression of USP50 is upregulated in bile reflux gastritis and GC. **(A)** Protein levels of USP50 in gastric tissues from Normal, BRG, and GC (gastric cancer) patients were detected by western blotting. **(B)** Protein levels of USP50 in murine gastric tissues of the Sham and GJ groups were assayed with western blotting. **(C)** The expression of USP50 was detected by IF in normal tissues and BRG tissues (red). CD68 was used as a marker for macrophages(green), and the cell nuclei were stained with DAPI (blue). White arrows indicate co-localization of USP50 and CD68. **(D)** The number of USP50 positive cells per field was counted in the IF of gastric tissues (field: 20 X). **(E)** Percentage calculation of co-localization between USP50 and CD68. The co-localization percentage is determined by dividing the number of cells positive for both USP50 and CD68 by the number of cells positive for USP50 in the same randomly selected field. (field: 20 X) **(F-I)** U937 and RAW264.7 cells were subjected to TDCA stimulation at varying doses or durations. qRT-PCR was performed to detect the mRNA level of USP50 **(F, G)**, with western blotting used to assay the protein level of USP50 **(H, I)**. *P < 0.05, **P < 0.01, and *** *P*<0.001.

The expression pattern of USP50 in bile reflux gastritis and GC has not been previously documented. Here, we validated the difference in the expression of USP50 in normal gastric mucosa, bile reflux gastritis, and GC. The data obtained from our cohort revealed that compared with normal subjects, patients with bile reflux gastritis and GC tissues presented gradual elevation of USP50 detected by western blotting (**Figure 2A**; **Supplementary Figure 2A**). In the inflammatory stomach of C57BL/6 mice undergoing GJ surgery, Western blotting and IHC both identified the considerably elevated expression of USP50 (**Figure 2B**; **Supplementary Figures 2B, C**). In the IF of gastric tissues, we found more USP50 positive cells were presented in BRG group (**Figures 2C, D**). Strikingly, we also found elevated co-expression percentage of USP50 and CD68+ macrophages in the gastric biopsies of bile reflux gastritis patients, in contrast to normal tissues (**Figures 2C, E**). On the other hand, this result also showed macrophages were the main cell type expressing USP50 in BRG. Additionally, in PMA-differentiated U937 and RAW264.7 macrophages, TDCA significantly enhanced the expression of USP50 at both mRNA and protein levels, following a pattern reliant on time or dosage (**Figures 2F-I**; **Supplementary Figures 2D, E**). Based on these findings, USP50 expression was elevated in bile reflux gastritis and GC.

### 3.3 USP50 accelerates bile acid-induced NLRP3 inflammasome activation

**Figure 3:**
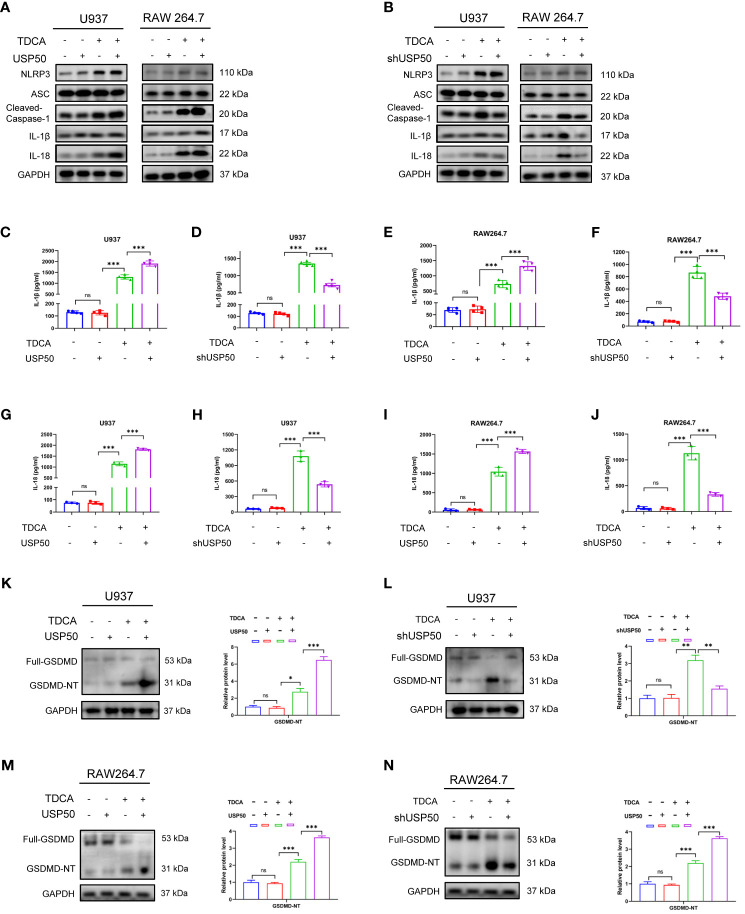
USP50 accelerates bile acid-induced NLRP3 inflammasome activation. **(A, B)** USP50 was overexpressed **(A)** or knock-down **(B)** in U937 and RAW264.7 cells and pyroptosis-related proteins were analyzed by western blotting. **(C-F)** IL-1β was measured by ELISA in U937 cells **(C, D)** and RAW264.7 cells **(E, F)** with or without USP50 modification. **(G-J)** IL-18 was measured by ELISA in U937 cells **(G, H)** and RAW264.7 cells **(I, J)** with or without USP50 modification. **(K, L)** Full-length GMDMD protein and GSDMD N-terminal (GSDMD-NT) were assessed by western blotting and quantified by Image J software in U937 cells with USP50 ectopic expression **(K)** or USP50-knockdown **(L)**. **(M, N)** Full-length GMDMD protein and GSDMD-NT were assessed by western blotting and quantified by Image J software in RAW264.7 cells with USP50 ectopic expression **(M)** or USP50-knockdown **(N)**. *P < 0.05, **P < 0.01, and *** *P*<0.001. "ns" means "no significance" (*P*>0.05).

The aforementioned findings indicate a potential scenario where USP50 could be a target influenced by signals from bile acids. We then sought to determine the role of USP50 on bile acid-induced NLRP3 inflammasome activation via USP50 loss- or gain-of-function experiments. Intriguingly, PMA-differentiated U937 and RAW264.7 cells with ectopic USP50 expression were more prone to the cleavage of caspase-1 and the maturation of IL-1β/IL-18 *in vitro* (**Figure 3A**; **Supplementary Figures 3C, D**). Consistently, USP50 depletion impeded the induction of caspase-1 cleavage and IL-1β/IL-18 maturation (**Figure 3B**; **Supplementary Figures 3E, F**). IL-1β (**Figures 3C-F**) and IL-18 (**Figures 3G-J**) levels measured by ELISA further corroborated the positive regulation of USP50 on bile acid-induced NLRP3 inflammasome activation.

The basic levels of the members of NLRP3 inflammasome were not affected by modulating USP50 expression without TDCA treatment. Intriguingly, upon DGR exposure, enhancing or depleting USP50 did not influence the inducing effect of DGR on NLRP3 inflammasome as well. GSDMD is the executor of pyroptosis. The production of GSDMD-N fragment induced by TDCA was enhanced or abrogated by USP50 overexpression (**Figure 3K**) or depletion (**Figure 3L**) in U937 cells. Similar results were found in RAW264.7 cells (**Figures 3M, N**). Consequently, these findings proposed the necessity of USP50 for the efficient initiation of NLRP3 inflammasome activation upon bile acid stimulation.

### 3.4 USP50 interacts with and deubiquitinates ASC to induce ASC speck formation and oligomerization

**Figure 4:**
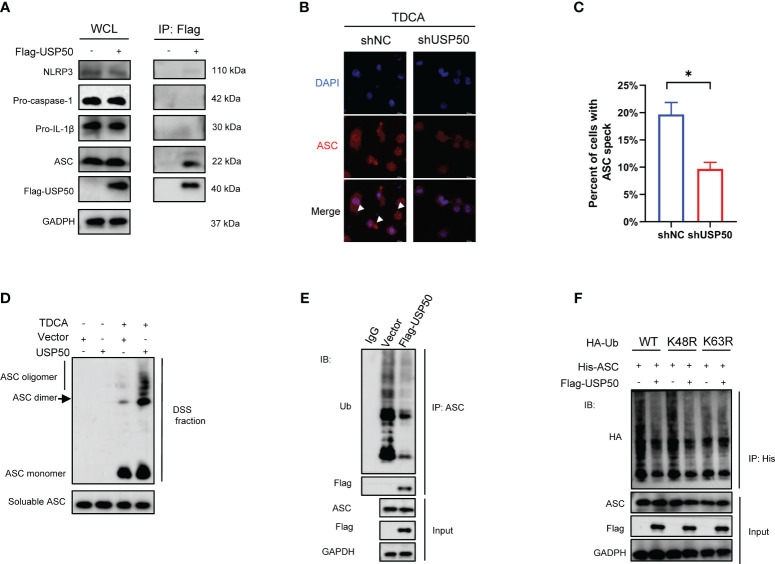
USP50 interacts with and deubiquitinates ASC to induce ASC speck formation and oligomerization. **(A)** Vector or Flag-USP50 was transferred to U937 cells and co-IP was performed with anti-Flag antibody. Whole-cell lysates (WCL) were used as Input. **(B)** ASC specks were detected by IF in U937 cells with or without USP50 knock-down. U937 cells were stimulated with TDCA (200μM) after being differentiated by PMA and primed by LPS. White arrowheads indicated ASC specks. **(C)** ASC specks of five random fields in each group were counted and analyzed with the student’s t-test. (*P<0.05). **(D)** Differentiated and primed U937 cells with or without USP50 overexpression were subjected to TDCA stimulation. DSS was used to crosslink ASC oligomers. Western blotting was utilized to analyze ASC aggregation. **(E)** U937 cells transfected with Vector or Flag-USP50 were lysed with IP lysis buffer and co-IP was conducted with anti-ASC antibody. Ub antibody was used to assay the ubiquitination of ASC by western blotting. MG132 (20μM) was added to the medium 6 hours before collecting cells. **(F)** WT, K48R, or K63R HA-Ub was co-transferred to 293T with His-ASC with or without Flag-USP50. co-IP was performed with His antibody and ubiquitination of ASC was analyzed with HA antibody via western blotting. MG132 was added to the culture medium 6 hours before collecting the cells.

The above findings motivate us to investigate whether USP50 modulates inflammasome activation based on its deubiquitinating enzyme function. Flag-USP50 was stable expressed in U937 cells via lentiviral transfection and then co-IP was conducted with anti-Flag antibody. According to co-IP results, USP50 exhibited a robust interaction with ASC (**Figure 4A**). However, it appeared to have a less pronounced interaction with NLRP3 as well (**Figure 4A**). On the contrary, USP50 did not exhibit binding interaction with procaspase-1 and pro-IL-1β (**Figure 4A**). The significant correlation between USP50 and the ASC protein implies that the ASC protein could potentially be targeted by USP50. The formation of ASC specks is crucial for the activation of caspase-1. Regulating the formation of ASC specks presents a novel avenue for the treatment and prevention of inflammasome-associated diseases. Subsequently, the abundance of ASC specks was evaluated. Compared with the control group, the quantities of ASC specks prompted by bile acids exhibited a noteworthy decrease following USP50 knockdown (**Figures 4B, C**). Western blotting revealed the formation of ASC oligomers was barely detectable without bile acids challenge compared with cells upon bile acid treatment, while USP50 overexpression was able to further elevate ASC oligomerization in macrophages (**Figure 4D**).

We further assessed the effect of USP50 on ASC ubiquitination. Expression of exogenous USP50 greatly decreased ASC ubiquitylation (**Figure 4E**). Then we attempted to identify the type of linkage used for the USP50-mediated deubiquitylation of ASC. Wild-type ubiquitin, along with K48R and K63R mutant ubiquitin variants, were employed to assess the capacity of USP50 in facilitating ASC deubiquitylation. The results indicated that the coexpression of ASC with Ub-K63R failed to result in deubiquitylation of ASC (**Figure 4F**). Thus, these results indicated that USP50 catalyzes predominantly the K63-linked ubiquitination of ASC. Taken together, these data suggested that USP50 physically interacted with and deubiquitinated ASC to induce ASC speck formation and oligomerization.

### 3.5 USP50 stimulates HMGB1 release in DGR-induced gastric inflammation

**Figure 5:**
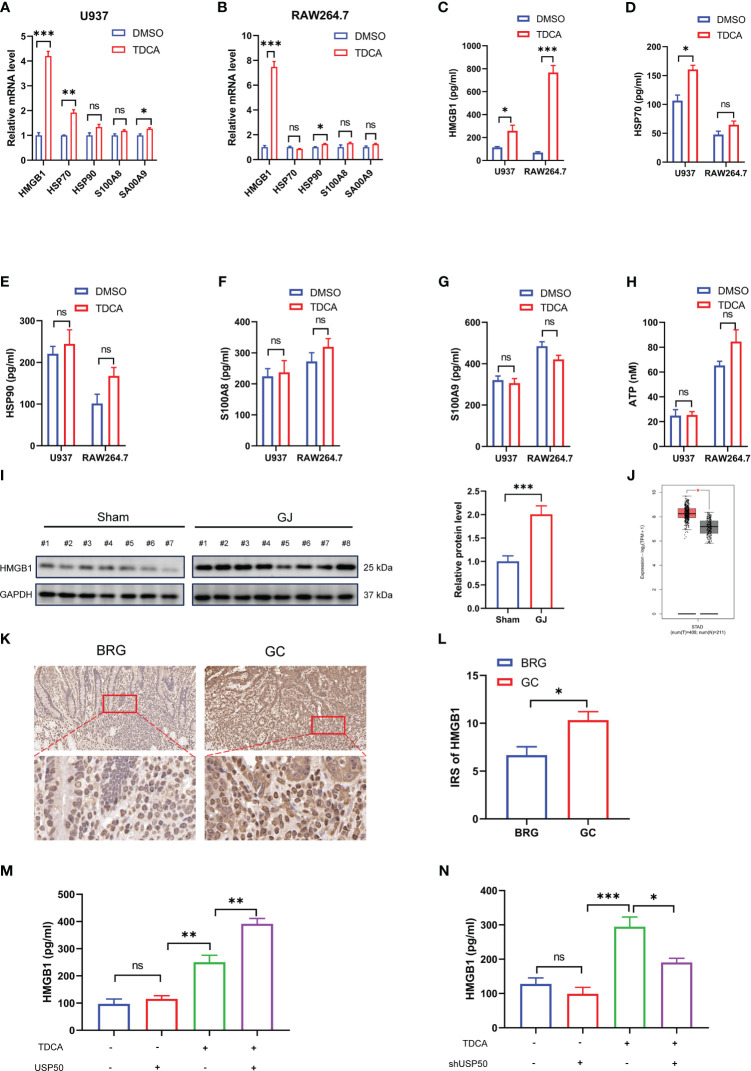
USP50 stimulates HMGB1 release in DGR-induced gastric inflammation. **(A, B)** mRNA levels of HMGB1, HSP70, HSP90, S100A8, and S100A9 were detected via qRT-PCR in U937 cells **(A)** or RAW264.7 cells **(B)** treated with DMSO or TDCA (200μM, 24 h). **(C-H)** ELISA was used to measure the concentrations of HMGB1 **(C)**, HSP70 **(D)**, HSP90 **(E)**, S100A8 **(F)**, and S100A9 **(G)** in the supernatant of U937 cells and RAW264.7 cells, while ATP concentration **(H)** was surveyed by ATP assay kit. **(I)** The protein level of HMGB1 was detected in gastric tissues of Sham and GJ mice by western blotting. Image J was used for quantitative analysis, with GAPDH as loading control. **(J)** The level of HMGB1 between normal gastric tissues and gastric cancer tissues was analyzed via GEPIA database (gepia.cancer-pku.cn/). **(K)** The IHC staining of HMGB1 was performed with sections from BRG patients and GC patients. **(L)** IRS was calculated for **(K)**. **(M, N)** HMGB1 was assayed by ELISA in the supernatant of U937 cells with USP50 overexpression **(M)** or knock-down **(N)**. *P < 0.05, **P < 0.01, and *** *P*<0.001. "ns" means "no significance" (*P*>0.05).

Inflammasome-mediated pyroptosis leads to the rupture of the cell membrane accompanied by the efflux of intracellular DAMPs, which may induce inflammatory cascades. A prior study suggested that DAMPs may play a role in carcinogenesis as well (24). Hence, we investigated whether DAMPs released from macrophages are involved in bile reflux gastritis progression. To identify the DAMPs changed in bile acids-induced pyroptosis, transcriptional levels of common DAMPs were detected by qRT-PCR first. The results revealed conspicuous elevated levels of HMGB1 in both U937 and RAW264.7 macrophages exposed to TDCA than in the DMSO-treated cells (**Figures 5A, B**). Given that extracellular HMGB1 is implicated in inflammation and cancer (25), ELISA was then applied to test the quantity of HMGB1 in the culture fluid. Consistently, a higher concentration of HMGB1 was detected in the supernatant of macrophages upon TDCA treatment (**Figure 5C**). Additionally, the results indicated a remarkable increase of HMGB1 but not HSP70, HSP90, S100A8, S100A9 or ATP released by both U937 and RAW264.7 macrophages with TDCA exposure (**Figures 5D-H**). In gastric biopsies of the DGR model undergoing GJ surgery, the markedly elevated HMGB1 was detected by western blotting (**Figure 5I**). In addition, we evaluated the level of HMGB1 in normal gastric tissues and gastric cancer tissues. The analysis from GEPIA database (gepia.cancer-pku.cn/) showed that HMGB1 is obviously elevated in gastric cancer tissues (**Figure 5J**). The IHC staining of gastric tissue sections from patients suggested that, HMGB1 is increased in the GC patients compared with BRG patients (**Figures 5K, L**). These results indicated HMGB1 may involve in the gastric tumorigenesis.

To verify the involvement of USP50 in the HMGB1 release, we subjected USP50-modified macrophages to the TDCA challenge. In the absence of TDCA, the extracellular HMGB1 concentrations were modest and exhibited no substantial variation between the control and USP50-modified cells (**Figures 5M, N**). However, upon TDCA exposure, ectopic expression of USP50 significantly increased HMGB1 release (**Figure 5M**), while USP50 depletion attenuated this release (**Figure 5N**). Collectively, these findings suggested that USP50 enhanced HMGB1 release in DGR-induced gastric inflammation.

### 3.6 USP50-HMGB1 axis contributes to DGR-induced gastric tumorigenesis

**Figure 6:**
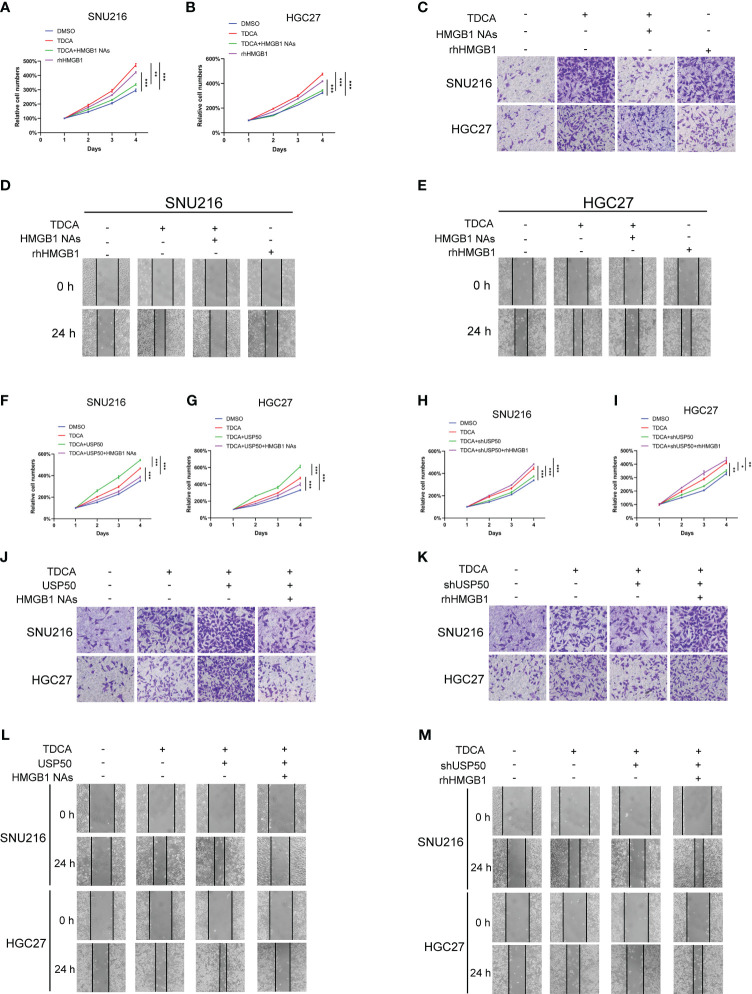
USP50-HMGB1 axis contributes to DGR-induced gastric tumorigenesis. CM (conditioned media) was obtained from the supernatant of DMSO (DMSO-CM) or TDCA (TDCA-CM) stimulated parental U937 cells or USP50-modified U937 cells. Gastric cell lines SNU216 and HGC27 were cultured in CM. **(A, B)** CCK-8 assay was performed to assess cell viability of SNU216 **(A)** and HGC27 cells **(B)** in various groups including DMSO (DMSO-CM), TDCA (TDCA-CM), TDCA+HMGB1 NAs (TDCA-CM with HMGB1 NAs added), and rhHMGB1 (rhHMGB1 added to media). **(C)** Cell invasion ability *in vitro* was tested via Transwell assay. **(D, E)** Scratch assay was implemented to evaluate the cell motility of SNU216 **(D)** and HGC27 **(E)**. **(F, G)** The role of CM from USP50-overexpressed U937 cells and HMGB1 NAs on cell viability of SNU216 **(F)** and HGC27 **(G)** were assayed with a CCK-8 kit. **(H, I)** The effect of CM from USP50 knock-down U937 cells and rhHMGB1 on cell viability of SNU216 **(H)** and HGC27 **(I)** was detected by the CCK-8 method. **(J, K)** Transwell assay of SNU216 and HGC27 cells. Gastric cells were cultured with CM from USP50-overexpressed **(J)** or USP50-knockdown **(K)** U937 cells added with HMGB1 NAs or rhHMGB1. **(L, M)** Scratch assay was performed to evaluate cell motility. Ditto, SNU216 and HGC27 were cultured in CM, added with HMGB1 NAs or rhHMGB1. *P < 0.05, **P < 0.01, and *** *P*<0.001.

In order to investigate the involvement of the USP50-HMGB1 axis in DGR-induced gastric tumorigenesis, gastric cancer cell lines SNU216 and HGC27 were co-cultured with conditioned media (CM) from macrophages exposed to TDCA (TDCA-CM). Subsequently, the resulting aggressive phenotype was examined. The TDCA-CM greatly facilitated tumor cell viability (**Figures 6A, B**), invasion (**Figure 6C**; **Supplementary Figure 4A**), and motility (**Figures 6D, E**; **Supplementary Figure 4B**) in contrast to the co-culture with the control, while HMGB1 neutralizing Abs antagonized this effect. Meanwhile, the addition of rhHMGB1 to the culture medium mimicked the inducing effect of this co-culture exposed to TDCA (**Figures 6A-E**; **Supplementary Figures 4A, B**).

Furthermore, enhancing USP50 expression in macrophages accelerated the promoting effect of TDCA-CM on the aggressive behavior of tumor cells, which was abolished by HMGB1 neutralizing Abs (**Figures 6F, G, J, L**; **Supplementary Figures 4C, E**). Depletion of USP50 in macrophages impaired the aggressive phenotypes triggered by the co-culture exposed to TDCA-CM. The tumor-inhibitory effect of USP50 depletion was reversed by rhHMGB1 (**Figures 6H, I, K, M**; **Supplementary Figures 4D, F**). These results suggest that the USP50-HMGB1 axis may contribute to gastric tumorigenesis.

### 3.7 USP50-HMGB1 axis promotes tumor proliferation and invasion through PI3K/AKT and MAPK/ERK pathways

**Figure 7:**
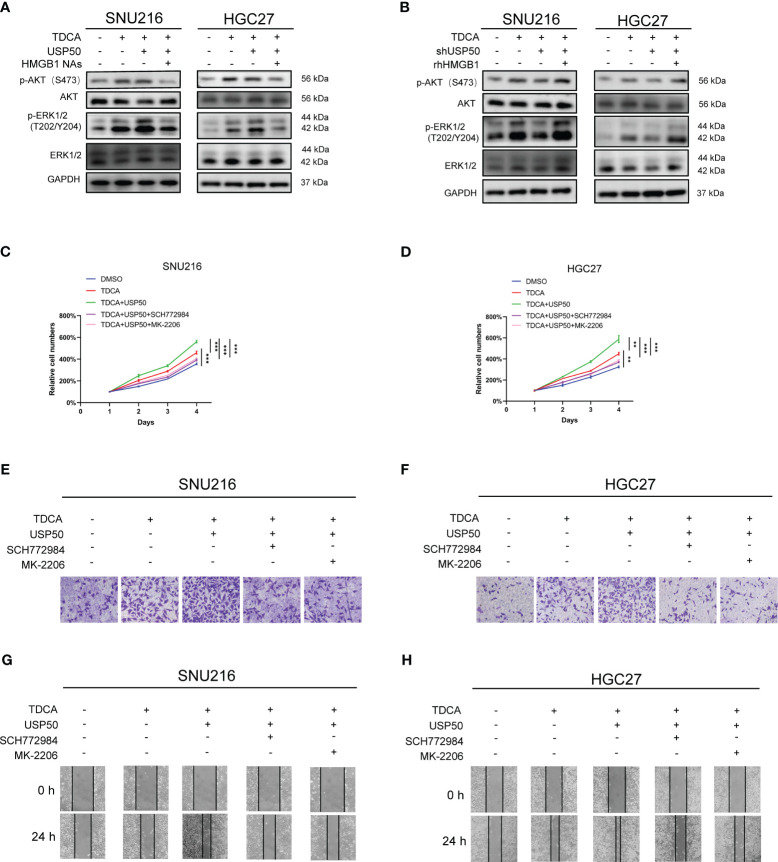
USP50-HMGB1 axis promotes tumor proliferation and invasion through PI3K/AKT and MAPK/ERK pathways. CM was collected from the supernatant of USP50-modified U937 cells (USP50 or shUSP50) with or without TDCA stimulation (TDCA). CM was applied to gastric cells combined with or without HMGB1 NAs or rhHMGB1. **(A)** The effect of CM derived from U937 cells in diverse situations and HMGB1 NAs on key proteins of PI3K/AKT and MAPK/ERK pathways was detected in SNU216 and HGC27 cells via western blotting. **(B)** The effect of CM derived from TDCA-challenged, with or without USP50-knockdown U937 cells, combined with HMGB1 NAs on key proteins of PI3K/AKT and MAPK/ERK pathways was detected in SNU216 and HGC27 cells via western blotting. **(C, D)** CM combined with or without ERK1/2 inhibitor (SCH772984) and an AKT inhibitor (MK-2206) was used to culture SNU216 **(C)** and HGC27 **(D)** cells. Cell viability was evaluated with CCK-8 kits. **(E-H)** The effect of various types of CM, SCH772984, and MK-2206 on cell invasion **(E, F)** and cell mobility **(G, H)** in SNU216 and HGC27 cells. **P < 0.01, and *** *P*<0.001.

Earlier research has indicated that obstructing the RAGE-HMGB1 axis curbs both tumor cell growth and metastasis through the inhibition of PI3K/AKT and MAPK/ERK pathway activation (26–28). Consequently, we evaluated the potential involvement of the PI3K/AKT and MAPK/ERK pathways in HMGB1-associated gastric tumorigenesis. We found that SNU216 and HGC27 cells co-cultured with TDCA-CM showed an increased presence of p-AKT (S473) and p-ERK1/2 (T202/Y204) compared to the DMSO-CM (**Figures 7A, B**; **Supplementary Figures 5A-D**). Enhancing USP50 expression augmented this inducing effect, but was attenuated by neutralizing anti-HMGB1 (**Figure 7A**; **Supplementary Figures 5A, B**). Depletion of USP50 in macrophages impaired the elevation of p-AKT (S473) and p-ERK1/2 (T202/Y204) induced by the co-culture exposed to TDCA-CM (**Figure 7B**; **Supplementary Figures 5C, D**). The inhibitory effect of USP50 depletion was abolished by rhHMGB1 treatment (**Figure 7B**; **Supplementary Figures 5C, D**). To confirm that USP50-HMGB1 axis-driven gastric tumorigenesis is mediated by the PI3K/AKT and MAPK/ERK pathways, herein we utilized ERK1/2 inhibitor SCH772984 and AKT inhibitor MK-2206. Results showed both compounds inhibited the enhanced proliferation and invasion ability of SNU216 and HGC27 cells co-cultured with TDCA-CM (**Figures 7C-H**; **Supplementary Figures 5E, F**). In summary, the USP50-HMGB1 axis facilitates gastric tumorigenesis through the PI3K/AKT and MAPK/ERK pathways.

## 4 Discussion

Bile reflux has long been bound up with the initiation of precancerous lesions and the development of gastric cancer. Interaction between BAs and gastric mucosa epithelial cells gives rise to gastric mucosal inflammation, which varies from duration and intensity of bile reflux. Beyond inducing inflammation, prolonged exposure to BAs can also result in intestinal metaplasia of the gastric mucosa, ultimately contributing to tumorigenesis. Although increasing studies propose that bile reflux serves as an initiating factor for gastric cancer occurrence, the exact mechanism remains elusive.

Numerous prior studies have concentrated on the direct harmful impact of bile acids on the gastric mucosa itself following BRG, while the influence of bile acids as DAMPs received less attention. In bile reflux gastritis, higher levels of bile acids were observed in the plasma (29), favoring the increased uptake of bile acids into macrophages. Hence, bile acids extend their role as lipid solubilizers, assuming an even more pivotal role as signaling molecules in regulating the immune system. Hao et al. (30) have verified that bile acids, in the context of cholestasis, activate the NLRP3 inflammasome. Herein, we presented a variety of evidence, employing both *in vitro* and *in vivo* models, to substantiate the involvement of bile acids in the activation of NLRP3 inflammasome from macrophages in bile reflux gastritis. Conversely, Guo et al. (31) documented that bile acids potentially suppress the NLRP3 inflammasome by activating TGR5. The functional impact of bile acids on the NLRP3 inflammasome appears to be subject to variation based on distinct immune cell types, bile acid species, and the concentrations tested.

Pyroptosis is a newly recognized form of programmed cell death linked to inflammation. Following inflammasome activation, caspases cleave and assemble Gasdermin family members like GSDMD, which can puncture cells and cause their demise. Direct cell membrane perforation and rupture caused by pyroptosis lead to the overwhelming release of proinflammatory factors. Therefore, pyroptosis has been commonly observed in a range of chronic inflammatory conditions and autoimmune diseases (32, 33). Nonetheless, there exists a scarcity of investigations pertaining to the involvement of pyroptosis in bile reflux gastritis, with its signaling mechanism remaining incompletely elucidated. The data we present herein, as far as we know, offers the initial unveiling of this aspect that macrophage pyroptosis contributes to gastric inflammation by releasing proinflammatory cytokines.

Under physiological conditions, the activation of the inflammasome in response to pathogens and host-derived damaging signals amplifies the body’s defensive capacity against detrimental stimuli, while excessive inflammasome activation results in inflammatory damage, even chronic inflammatory-related cancer (10). Hence, the activation of the inflammasome is subject to multiple tight regulation. As expected, a set of inhibitory regulators for the NLRP3 inflammasome were discovered. IKKα negatively regulates inflammasome activation via IKKi-IKKα-ASC axis (34). In addition to modulation with ASC, NLRP3 also acts as a substrate for regulating inflammasome activation. Aryl hydrocarbon receptor exerts a negative regulation on NLRP3 inflammasome activity by suppressing NLRP3 at transcriptional level (35). Despite these strides in comprehending the negative regulation of inflammasomes, a comprehensive understanding of the causal connection between these regulators and the pathological progression of bile acids-related diseases is still limited. MFN2 mediated bile acid activation of inflammasome through mediating calcium transfer from the ER to mitochondria (15). Hao et al. (30) found bile acids trigger an extended calcium influx and work in concert with ATP, resulting in the activation of NLRP3 inflammasome, whereas FXR enacts negative regulation on the NLRP3 inflammasome through direct physical interaction with NLRP3 and caspase 1. In the current study, to the best of our understanding, our data revealed for the first instance that the NLRP3 inflammasome is significantly post-translationally regulated by USP50 in promoting BRG. Mechanically, USP50 physically interacts with ASC, reducing its K63-linked ubiquitination and enhancing ASC oligomerization, subsequently promoting the assembly and activation of the NLRP3 inflammasome. Consistently, the downregulation of USP50 distinctly alleviated bile acids-induced pyroptosis in macrophages. These findings strongly highlight the significance of the bile acid-USP50 axis in regulating the NLRP3 inflammasome in BRG.

Damage-associated molecular patterns (DAMPs) are molecules that are secreted, released, or exposed on the cell surface by cells undergoing death, stress, or injury, which include HMGB1, S100A8, S100A9, HSP70, HSP90, ATP, IL-1α, IL-33 and so on (36). Released DAMPs may cause aberrant inflammation (37), while this sterile inflammation may lead to the development of numerous inflammatory diseases, such as metabolic disorders, neurodegenerative diseases, autoimmune diseases, and cancer (38). Especially, chronic inflammation has been proved to promote tumorigenesis and metastasis of gastric cancer (39). Previous studies revealed cells undergo pyroptosis with their plasma membrane rupture, leading to the release of various molecules, including DAMPs (40). In our investigation, we discovered that the application of bile acids could lead to a notable enhancement in the expression and subsequent release of HMGB1, rather than other DAMPs both in the BRG murine model and cellular models *in vitro*.

HMGB1 is non-histone, nuclear DNA-binding protein regulates transcription, and is involved in organization of DNA. A growing body of research finds that, in addition to its architectural function in the nucleus, HMGB1 is also present in the cytosol and can be secreted or released from cells (41, 42). The subcellular localization (in the nucleus, cytosol, cell membrane and extracellular space) of HMGB1 plays a pivotal role in defining its functionality and its interaction with various cellular constituents. HMGB1 can not only be actively secreted by immune cells and other cell types, but also be released passively by all cell types upon cell death, including pyroptosis (43). Toll-like receptor 4 (TLR4) and the advanced glycosylation end product-specific receptor (AGER; also known as RAGE) are the most well-studied receptors for extracellular HMGB1, binding of which leads to the activation of downstream signaling pathways. HMGB1 plays a crucial role in various cellular processes (41). HMGB1 not only participates in infectious inflammation (such as sepsis), but also involves in sterile inflammation (such as strokes, acute myocardial infarction) (25). The role of HMGB1 in cancer is intricate, with implications for tumor development, progression, metastasis, and the response to chemotherapeutics involving both intracellular/nuclear and extracellular forms of HMGB1. Tan et al. (24) found HMGB1 participates in the tumorigenesis of colorectal cancer through the ERK1/2 pathway. Recent studies also found exogenous HMGB1 participated in the epithelial-mesenchymal transition via the PI3K/Akt/mTOR pathway in pulmonary fibrosis (44). Another study showed that HMGB1-RAGE through PI3K/AKT signaling promotes not only breast cancer cell invasion but also PD-L1 expression which leads to the destruction of the effector T cells (27). These researches indicated that extracellular HMGB1 could activate PI3K/AKT and MAPK/ERK pathways, which is consistent with current study. Our study also found HMGB1 release can be enhanced by overexpression of USP50 in macrophages with bile acids stimulated. Moreover, by investigating the function of HMGB1 in facilitating the development of gastric cancer, our results indicate that HMGB1 could potentially emerge as an innovative and appealing target for prospective clinical interventions aimed at treating gastric cancer.

## Data availability statement

The original contributions presented in the study are included in the article/**Supplementary Material**. Further inquiries can be directed to the corresponding authors.

## Ethics statement

All animal experiments in our study were carried out in accordance with the Helsinki Declaration, and approved by the Ethics Committee of The First Affiliated Hospital of Xi’an Jiaotong University. Patients were informed that the resected specimens were stored by the hospital and potentially used for scientific research, and that their privacy would be maintained. All patients provided informed consent prior to undergoing surgery. Our study protocol was approved by the Ethics Committee of the First Affiliated Hospital of Xi’an Jiaotong University.

## Author contributions

CZ: Conceptualization, Data curation, Investigation, Methodology, Visualization, Writing – original draft. MM: Conceptualization, Data curation, Formal Analysis, Investigation, Writing – original draft. XL: Data curation, Formal Analysis, Investigation, Methodology, Writing – original draft. ZD: Investigation, Writing – original draft. JW: Investigation, Writing – original draft. CY: Data curation, Investigation, Writing – original draft. JZ: Data curation, Formal Analysis, Writing – original draft. XS: Conceptualization, Investigation, Supervision, Writing – review & editing. JY: Conceptualization, Funding acquisition, Project administration, Supervision, Validation, Writing – review & editing.
