# Supplementary material for: USP50 regulates NLRP3 inflammasome activation in duodenogastric reflux-induced gastric tumorigenesis
Source: Front Immunol. 2024 Feb 26;15:1326137. doi: 10.3389/fimmu.2024.1326137 (PMC10925683; doi:10.3389/fimmu.2024.1326137)
Supplement: Supplementary file 1 [file DataSheet_1.zip › All Essential Supplementary Materials/Table 1.DOCX]

| Gene name | Species | Primer sequence (5'-3') |
| --- | --- | --- |
| GAPDH-F | Human | TGCACCACCAACTGCTTAGC |
| GAPDH-R | Human | GGCATGGACTGTGGTCATGAG |
| USP50-F | Human | CCCATCAGCCTCCTTAACTG |
| USP50-R | Human | AAATGACTTCTCAGCCGTCTC |
| IL-1β-F | Human | CCAAAGAAGAAGATGGAAAAGC |
| IL-1β-R | Human | GGTGCTGATGTACCAGTTGGG |
| IL-18-F | Human | TCTTCATTGACCAAGGAAATCGG |
| IL-18-R | Human | TCCGGGGTGCATTATCTCTAC |
| HMGB1-F | Human | TATGGCAAAAGCGGACAAGG |
| HMGB1-R | Human | CTTCGCAACATCACCAATGGA |
| HSP70-F | Human | AGCTGGAGCAGGTGTGTAAC |
| HSP70-R | Human | CAGCAATCTTGGAAAGGCCC |
| HSP90-F | Human | TCTATTTGTCCCACGACG |
| HSP90-R | Human | ATCCTCCGAGTCTACCAC |
| S100A8-F | Human | ATGCCGTCTACAGGGATGAC |
| S100A8-R | Human | ACTGAGGACACTCGGTCTCTA |
| S100A9-F | Human | GGTCATAGAACACATCATGGAGG |
| S100A9-R | Human | GGCCTGGCTTATGGTGGTG |

**Primer for qRT-PCR**

| Gene name | Species | Primer |
| --- | --- | --- |
| GAPDH-F | Mouse | GTGGAGTCATACTGGAACATGTAG |
| GAPDH-R | Mouse | AATGGTGAAGGTCGGTGTG |
| USP50-F | Mouse | CATTCACTCTTCTTCGGCAGT |
| USP50-R | Mouse | GGCAGTCTTTACCCAGCTTT |
| HMGB1-F | Mouse | CTTTTGTCCACATGCCCTGC |
| HMGB1-R | Mouse | CCCACGGTGTGACAGTATTGA |
| HSP70-F | Mouse | TGCGTGGGCGTGTTCCA |
| HSP70-R | Mouse | GGTGTTCTGCGGGTTCAGC |
| HSP90-F | Mouse | ACGATTGGGAAGAACACT |
| HSP90-R | Mouse | GAAATTCAGATACTCAGGGA |
| S100A8-F | Mouse | TGCCCTCTACAAGAATGA |
| S100A8-R | Mouse | CACCATCGCAAGGAACT |
| S100A9-F | Mouse | GGTGGAAGCACAGTTGG |
| S100A9-R | Mouse | CATGATGTCATTTATGAGGG |
