# Supplementary material for: USP50 regulates NLRP3 inflammasome activation in duodenogastric reflux-induced gastric tumorigenesis
Source: Front Immunol. 2024 Feb 26;15:1326137. doi: 10.3389/fimmu.2024.1326137 (PMC10925683; doi:10.3389/fimmu.2024.1326137)
Supplement: Supplementary file 1 [file DataSheet_1.zip › All Essential Supplementary Materials/Table 2.DOCX]

**Supplementary Figure legends**

Fig. S1 Macrophage pyroptosis occurred in human bile reflux gastritis and experimental models.  **(A)** NLRP3, Caspase-1, and IL-1β were detected by IHC in mice from the Sham and GJ group. Scale bar, 100 µm (left), 50 µm (right). The immunoreactive score (IRS) of IHC was calculated and compared between the two groups by Student’s T test. **(B)** Pyroptosis-related proteins in the western blotting of Fig. 1K were quantified by Image J software and GAPDH was used as reference. **(C, D)** Quantification of western blotting in Fig. 1L, which showed the effect of different concentrations (stimulated for 24h) and durations (100μM TDCA) of TDCA on U937. **(E, F)** Quantification of western blotting in Fig. 1M.

Fig. S2 The expression of USP50 is upregulated in bile reflux gastritis and GC. **(A, B)** Relative level of USP50 in western blotting of Fig. 2A **(A)** and Fig. 2B **(B)** was quantified. **(C)** USP50 was stained by IHC in gastric tissues from Sham and GJ mice. **(D, E)** Quantification of western blotting in Fig. 2H **(D)** and Fig. 2I **(E)**.

Fig. S3 USP50 accelerates bile acid-induced NLPR3 inflammasome activation. **(A, B)** Western blotting was utilized to verify the effect of USP50 knock-down or overexpression in U937 **(A)** and RAW264.7 **(B)** cells. **(C, D)** Quantification of western blotting that modification of USP50 affected TDCA stimulated pyroptosis, which was presented in Fig. 3A. **(E, F)** Quantification of western blotting in Fig. 3B

Fig. S4 USP50-HMGB1 axis contributes to DGR-induced gastric tumorigenesis. **(A)** Quantification and statistical analysis of cells passed through transwell membrane in Fig. 6C. **(B)** Quantification and statistical analysis of wound healing assay in Fig. 6D-E. **(C, D)** Quantification and statistical analysis of transwell assay in Fig. 6J **(C)** and Fig. 6K **(D)**. **(E, F)** Quantification by Image J software and statistical analysis of wound healing assay were performed for Fig. 6L **(E)** and Fig. 6M **(F)**.

Fig. S5 USP50-HMGB1 axis promotes tumor proliferation and invasion through PI3K/AKT and MAPK/ERK pathways. **(A, B)** Quantification of western blotting of Fig. 7A concerning SNU216 **(A)** and HGC27 **(B)**. **(C, D)** Quantification of western blotting of Fig. 7B concerning SNU216 **(C)** and HGC27 **(D)**. **(E)** Quantification and statistical analysis of transwell assay in Fig. 7E and 7F. **(F)** Quantification and statistical analysis of wound healing assay in Fig. 7G and 7H.
