# Supplementary material for: USP50 regulates NLRP3 inflammasome activation in duodenogastric reflux-induced gastric tumorigenesis
Source: Front Immunol. 2024 Feb 26;15:1326137. doi: 10.3389/fimmu.2024.1326137 (PMC10925683; doi:10.3389/fimmu.2024.1326137)
Supplement: Supplementary file 1 [file DataSheet_1.zip › All Essential Supplementary Materials/Table 3.DOCX]

**Caption of Graphical Abstract**

This study revealed that elevated USP50 induced by BAs enhances NLRP3 inflammasome activation through deubiquitination of ASC, subsequently leading to macrophage pyroptosis and the release of HMGB1. HMGB1, in turn, promotes duodenogastric reflux-induced gastric tumorigenesis via the PI3K/AKT and MAPK/ERK pathways.
