# Supplementary figures and images for: USP50 regulates NLRP3 inflammasome activation in duodenogastric reflux-induced gastric tumorigenesis
Source: Front Immunol. 2024 Feb 26;15:1326137. doi: 10.3389/fimmu.2024.1326137 (PMC10925683; doi:10.3389/fimmu.2024.1326137)

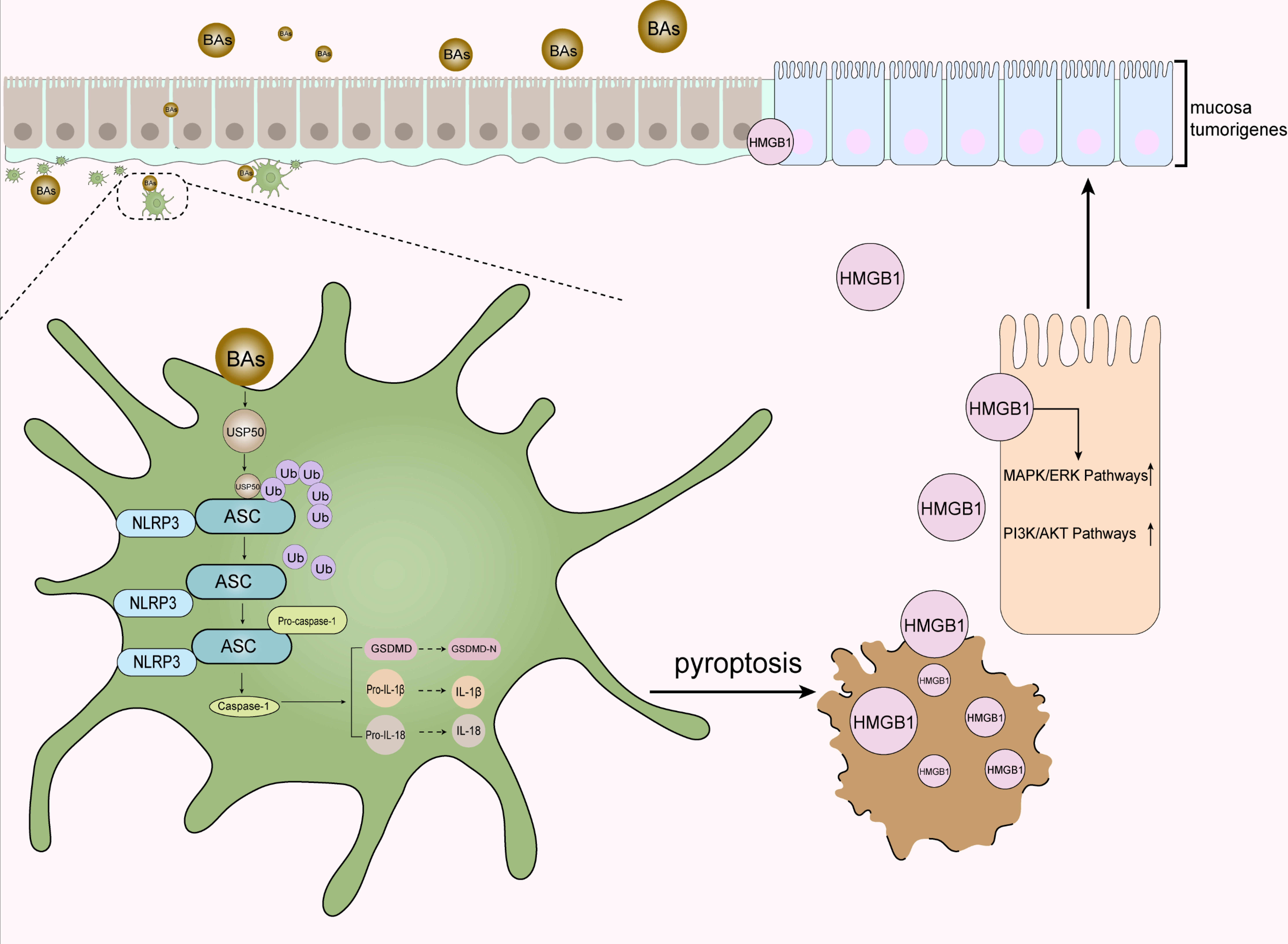

Supplement: Supplementary file 1 [file DataSheet_1.zip › All Essential Supplementary Materials/Image 6.tif]
